# Transcranial direct current stimulation in neglect rehabilitation after stroke: a systematic review

**DOI:** 10.1007/s00415-022-11338-x

**Published:** 2022-09-22

**Authors:** B. González-Rodriguez, N. Serradell-Ribé, R. Viejo-Sobera, J. P. Romero-Muñoz, Elena M. Marron

**Affiliations:** 1 Brain Damage Unit, Beata María Ana Hospital, Madrid, Spain; 2 Universidad Nacional de Educación a Distancia (UNED), Madrid, Spain; 3 Faculty of Health Sciences, Cognitive NeuroLab, Universitat Oberta de Catalunya, Madrid, Barcelona, Spain; 4 Faculty of Experimental Sciences, Universidad Francisco de Vitoria, Madrid, Spain

**Keywords:** Neglect, Non-invasive brain stimulation, Stroke, Transcranial electric stimulation, Transcranial direct current stimulation, tDCS

## Abstract

Hemispatial neglect is one of the most frequent attention disorders after stroke. The presence of neglect is associated with longer hospital stays, extended rehabilitation periods, and poorer functional recovery. Transcranial direct current stimulation (tDCS) is a new technique with promising results in neglect rehabilitation; therefore, the objective of this systematic review, performed following the PRISMA guidelines, is to evaluate the effectiveness of tDCS on neglect recovery after stroke. The search was done in MEDLINE (PubMed), Web of Science, Scopus, Cochrane Library, and BioMed Central databases. A total of 311 articles were found; only 11 met the inclusion criteria, including 152 post-stroke patients in total. Methodological quality and risk of bias were assessed for all the studies, and methodological characteristics of the studies, sample sizes, methods, main results, and other relevant data were extracted. tDCS intervention ranged from one to twenty sessions distributed in 1 day to 4 weeks, with intensity ranged from 1 to 2 mA. We found moderate evidence for the efficacy of tDCS in the rehabilitation of hemispatial neglect after a stroke, being more effective in combination with other interventions. Nonetheless, the limited number of studies and some studies' design characteristics makes it risky to draw categorical conclusions. Since scientific evidence is still scarce, further research is needed to determine the advantage of this treatment in acute, sub-acute and chronic stroke patients. Future studies should include larger samples, longer follow-ups, and broader neurophysiological assessments, with the final aim of establishing the appropriate use of tDCS as an adjuvant intervention in neurorehabilitation settings.

**Supplementary Information:**

The online version contains supplementary material available at 10.1007/s00415-022-11338-x.

## Introduction

Hemispatial neglect is one of the most frequent attention disorders after suffering a stroke, reaching an incidence between 25 and 50% [22]. Neglect is characterized by the difficulty or inability to detect, orient, and respond toward stimuli presented in the contralesional hemifield, and even to attend or recognize the part of one's own body contralateral to the injury [9, 18, 61, 62]. Neglect could include sensory, representational, and motor symptoms [10, 62], it can affect different frames of references (egocentric, allocentric) [14, 51], and different ranges of space (personal, peripersonal and extrapersonal) [2, 65]. In clinical practice, neglect subtypes usually overlap, and patients present mixed symptomatology [31]. Neglect is more prevalent and severe after right hemisphere stroke than after left insults [7, 13, 32, 60], with a prevalence between 15 and 75% in right damage, and 2–12% after left stroke [16].

Functional implications of neglect are very significant, although they vary depending on its severity, causing difficulties in the recovery of the patient’s independence, due to the impact on both basic and instrumental activities of daily life (i.e., cleaning, dressing, eating, money management, public transportation, etc.) [19, 37]. The presence of neglect is associated with longer hospital admissions, lower probability of returning home after hospital discharge, extended rehabilitation periods, and higher percentage of falls [15, 16, 30, 70, 71]. Furthermore, the presence of neglect is related to slower and poor functional recovery [13, 36, 37, 39].

Spontaneous recovery of neglect symptoms after stroke occurs in the acute and subacute stages in around two thirds of patients [38]. However, in approximately 40% of the cases, neglect symptoms persist after the first months following the insult [55], becoming chronic in around 35–50% of the patients [38], and therefore, imposing an extra burden on patient caregivers [13].

Nowadays, the available therapeutic approaches for neglect rehabilitation, including prism adaptation, visuospatial training, mental imagery therapy, space remapping, optokinetic stimulation, trunk rotation, limb activation, eye patching, video feedback training, vestibular stimulation, or neck muscle vibration, among others, have limited results, since their clinical effectiveness in terms of long-lasting functional improvement is not clear [3, 24, 48, 73]. The persistence of symptomatology and the impact it has on patients' independence, make it essential to further investigate to develop novel treatments that target the underlying dysfunctions of neglect appropriately. In this regard, interventions based on non-invasive brain stimulation techniques constitute a promising therapeutic approach.

Non-invasive brain stimulation techniques allow modulating brain activity in a safe and comfortable way. There is extensive empirical evidence confirming the capacity of these techniques to modulate brain activity, by increasing or decreasing the excitability of the cerebral cortex [59], and to achieve long-term effects [35]. The most widely used NIBS techniques are transcranial direct current stimulation (tDCS) and transcranial magnetic stimulation (TMS).

tDCS allows modulation of cortical activity by applying a low-intensity electrical current (between 1 and 2 mA, usually), placing two or more electrodes over the scalp. The electrical current flows between the electrodes (i.e., from anode to cathode), increasing the cortical excitability below the anode, and decreasing it below the cathode. Brain activity modulation through tDCS is achieved by influencing the action potential threshold, making it higher causing it to increase (cathodal stimulation) or decrease (anodal stimulation) without reaching an action potential [28].

**Fig. 1:**
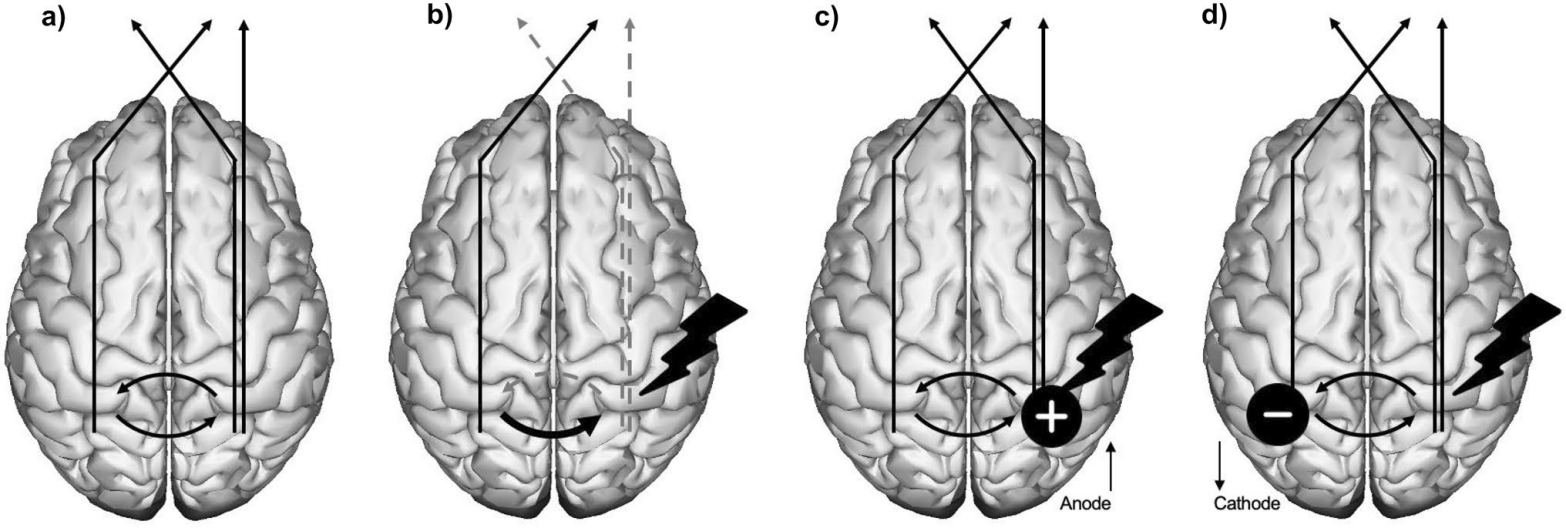
Excitatory and inhibitory tDCS protocols for neglect rehabilitation. **a** Dynamic balance between cerebral hemispheres through reciprocal excitatory and inhibitory transcallosal action. The right hemisphere regulates attention towards both hemi-fields, while the left hemisphere only regulates it towards the right hemi-field; that explains the higher incidence of neglect after right insults. **b** Unilateral brain damage breaks the balance: the affected hemisphere becomes hypoactive due to the damage, and the intact hemisphere becomes pathologically hyperactive. Neglect symptomatology is caused by both hypoactivity of the damaged hemisphere and hyperactivity of the intact one. **c** Excitatory tDCS protocols, aimed at increasing the activity of the damaged hemisphere, and **d** inhibitory tDCS protocols, to reduce the activity in the intact hemisphere. Both protocols can be applied to restore inter-hemispheric balance, and thus, alleviate neglect symptoms

The use of noninvasive brain stimulation for neglect rehabilitation is based on the Interhemispheric Rivalry Model, proposed by Kinsbourne in the past century [41–44]. Cerebral hemispheres are, at rest, in constant interaction, exerting a reciprocal inhibitory action through the existing transcallosal networks, maintaining a dynamic balance between them. According to the rivalry model, unilateral brain damage breaks this interhemispheric balance (see Fig. 1b). After the damage, the affected hemisphere becomes hypoactive, and therefore, it is not able to effectively inhibit the preserved hemisphere, making it hyperactive. This results in (a) pathological hyperactivity of the intact hemisphere (due to the absence of inhibition exerted over it by the damaged hemisphere), and (b) hypoactivity of the affected hemisphere because of the damage and the greater inhibition exerted on it by the intact hemisphere. Thus, the underlying dysfunction of neglect is both hypoactivity of the damaged hemisphere and pathological hyperactivity of the intact one. Based on this model, excitatory (aimed at increasing the activity of the damaged hemisphere) and inhibitory (to reduce the activity in the intact hemisphere) tDCS protocols are applied to restore the interhemispheric balance (see Fig. 1c, d).

Some studies have proven that tDCS can be an effective technique as a complementary therapeutic approach to more conventional treatments after stroke [21], showing promising results in the rehabilitation of visuospatial neglect (e.g., [5, 47, 74]). Here we performed a systematic review to gather the up-to-date evidence of the potential of tDCS as a novel intervention approach for neglect recovery after stroke, either in isolation or as an adjuvant approach to other treatments.

## Materials and methods

This systematic review was performed according to the Preferred Reporting Items for Systematic Reviews and Meta-Analyses (PRISMA) guidelines [53].

The protocol was registered at the International Prospective Register of Systematic Reviews (PROSPERO), on 31 July 2021, under identification number CRD42021255703, and can be accessed online (https://www.crd.york.ac.uk/prospero/display_record.php?ID=CRD42021255703).

### Identification and selection of studies

A systematic search in electronic databases (up to January 2021) including MEDLINE (accessed through PubMed), SCOPUS, Cochrane Central Register of Controlled Trials (Cochrane CENTRAL), and BioMedCentral was performed. In addition, we hand-searched the references of included studies to identify other relevant research. We also tried to identify unpublished studies or ongoing trials by searching clinical trial registries (ClinicalTrials.gov). The complete search strategy can be consulted in Supplementary material.

After removing duplicates, two reviewers (NSR and BGR), separately and independently, reviewed the titles and abstracts of the identified studies, and duplicates were eliminated. For the study selection, we included randomized controlled trials, crossover trials, and single case studies focused on determining the effects of tDCS as a treatment of hemineglect secondary to stroke, combined or not with other therapies. Disagreements were resolved by consensus or by a third reviewer (EMM). Once the final set of studies was selected, the same authors independently extracted the relevant data: methodological characteristics of the studies, the number of participants, comparison groups, interventions, and results, using standardized spreadsheets. Disagreements on data extracted were resolved by consensus or by a third reviewer (EMM).

### Eligibility criteria

Only articles in English or Spanish, and published in peer-review journals were included. The articles were selected based on the research question, elaborated following the PICOS model: (1) Patients with hemispatial neglect, with or without comorbidities. When the studies included data about multiple neurological conditions, only the results related to neglect were considered. (2) Study type: experimental studies, pilot studies and case reports. (3) Intervention: interventions studying the effect of tDCS in the rehabilitation of neglect, alone or combined with other techniques. (4) Population: adults with neglect.

### Risk of bias and methodological quality assessment

Methodological quality and risk of bias were assessed for all the studies. To evaluate the risk of bias, the two reviewers independently assessed each study using the Cochrane Collaboration’s tool [33]. This tool includes five domains: selection bias, performance bias, detection bias, attrition bias, and reporting bias. Each item is classified as a low (green), unclear (yellow), or high (red) risk of material bias.

The assessment of the methodological quality of the trials was carried out using the PEDro scale (www.pedro.org.au). This scale is made up of 11 items. Each item (except item 1, which relates to the external validity of the test), contributes 1 point to the total score, in a range of 0–10 [49]. In addition, studies were classified as follows regarding their methodological quality (see Ref. [23]: scores 9 or 10 = excellent, 6–8 = good; 4 or 5 = fair; < 4 = poor.

All the items of PEDro scale and Cochrane Collaboration’s tool were assessed by two reviewers independently (N.S.R and B.G.R.). One discrepancy appeared in the Cochrane tool, and it was discussed, including a third reviewer (E.M.M.) guiding the discussion, until reaching 100% agreement.

## Results

**Fig. 2:**
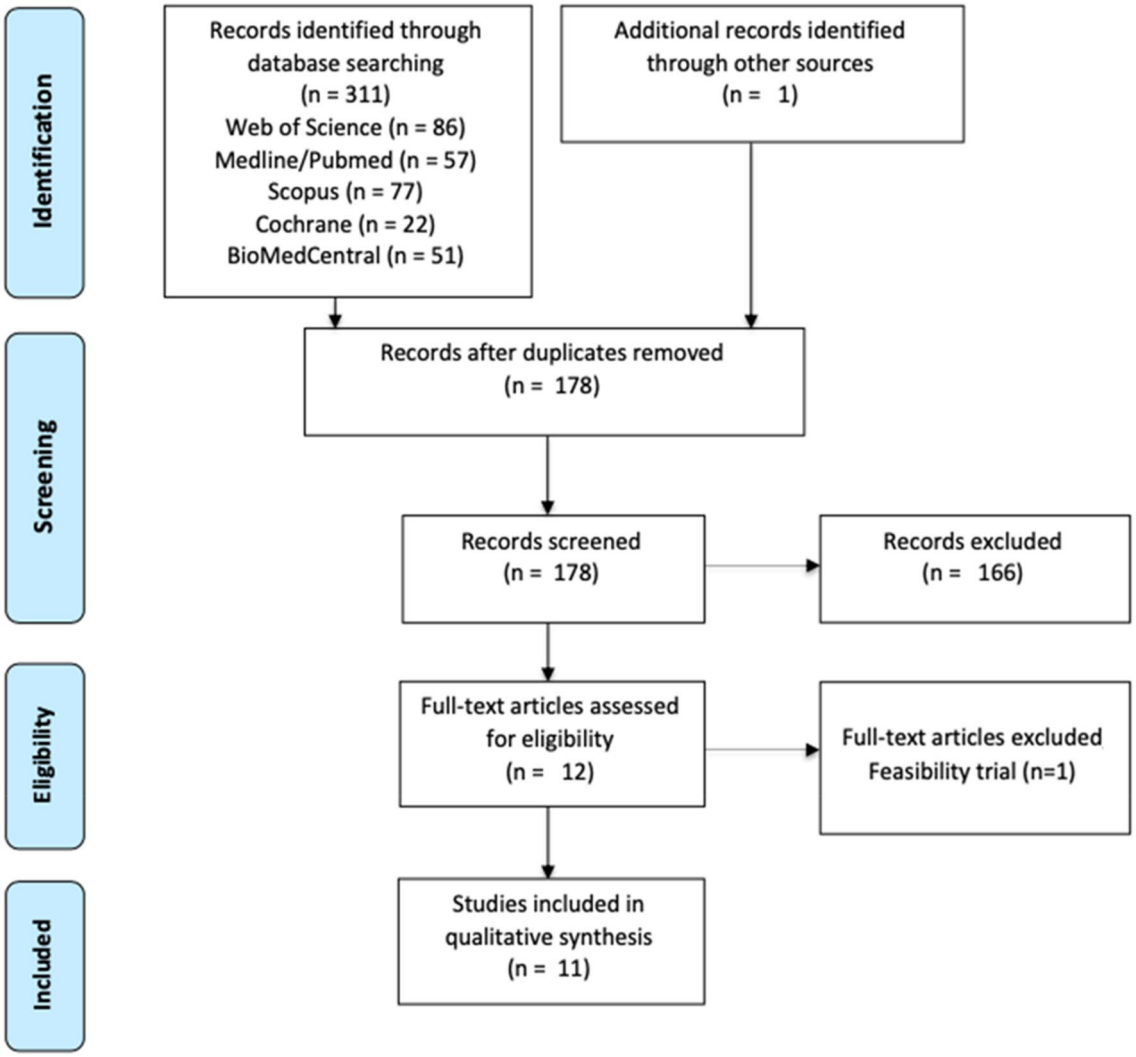
Study selection diagram flow

From a total of 311 studies initially identified, only eleven were considered for a qualitative synthesis (Fig. 2).

### Selection process and data extraction

The combined search of Web of Science, MEDLINE (PubMed), Scopus, Cochrane Library, and BioMed Central provided a total of 311 documents. The search strategy is included in the Supplementary material. An article identified through other sources was also included. After discarding duplicates, 178 documents remained. Of these, 166 records were excluded after reviewing the abstract, because they did not meet the inclusion criteria. The main reasons for exclusion were: having recruited a clinical sample without neglect, not being an experimental or case report article (being protocols, reviews, book chapters, etc.), not using tDCS. Most of the papers excluded satisfied more than one exclusion criteria, and none were excluded for not being written in English or Spanish. Although 12 articles made it to the eligibility phase, we had to discard one of them for being a feasibility trial with no analysis of the results. Studies relevant to the topic but not published in peer-reviewed journals, such as conference posters and abstracts, were not considered. Ultimately, a total of 11 studies were included in the review (see flow diagram in Fig. 2).

### Synthesis of results

Since there is much information extracted from the studies included in the review, the authors provide a different table for each section in Supplementary material (Tables S1–S4) and a comprehensive summary table with the main studies data at the end of the article (Table 3).

### Study goals, design, and methodology

The studies can be grouped into three major categories: (a) studies focused on assessing the efficacy of tDCS in neglect recovery in isolation (four studies), (b) efficacy of tDCS in combination with other specific neglect intervention (four studies), and (c) tDCS and conventional treatment jointly (three studies).

The articles reviewed included four parallel and seven crossover studies. Three were case reports, while eight used groups of patients. Regarding the randomization, six were randomized, and five were either not randomized or it was not specified by the authors. Six studies were double-blind, and five were not blind, or it was not specified.

Four studies used a control group, while seven used within-subject measures. In eight cases, sham was used as control; one study combined two different controls, sham and prism adaptation alone, and two studies used the standard treatment alone as control.

### Risk of bias and methodological quality

**Fig. 3:**
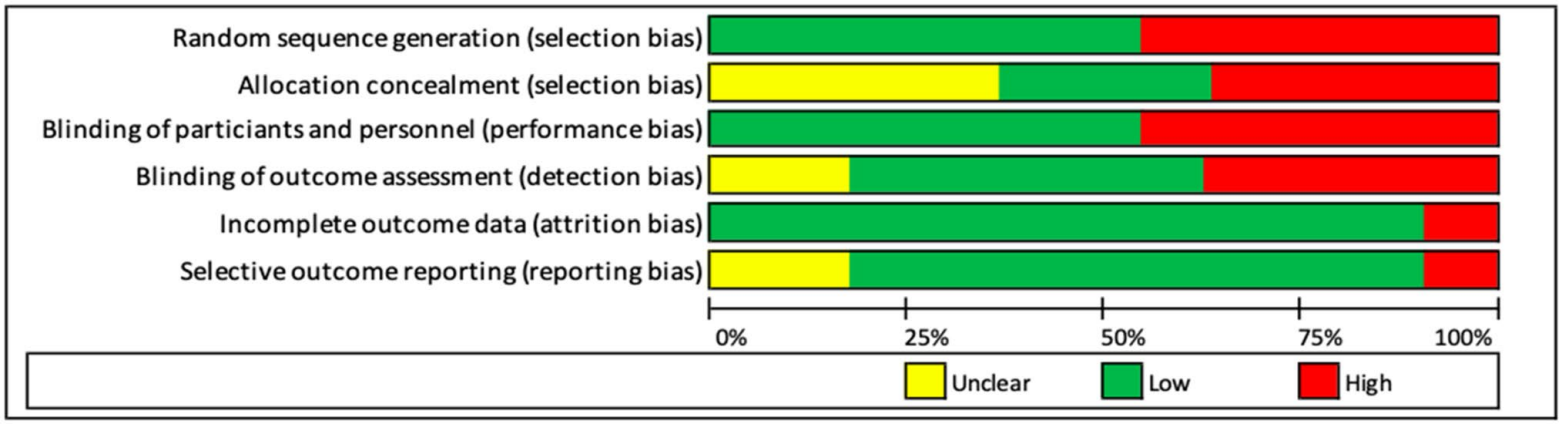
Risk of bias summary (percentage)

The risk of bias in the studies included in our review can be found in Table 1 (detailed information) and Fig. 3 (summary).

**Table 1:**
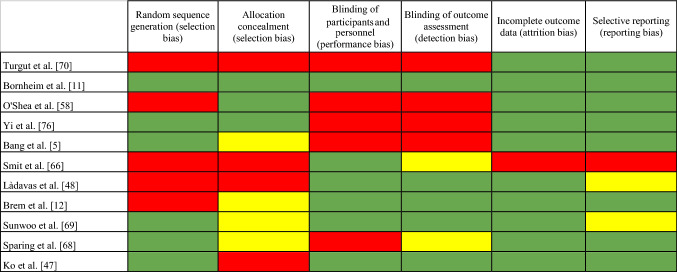
Risk of bias of the studies included in the systematic review

The selection bias reflected a great divergence between the studies. Six studies (54.5%) were classified as low risk, while five (45.5%) were considered high risk in Random sequence generation. In Allocation concealment, the differences are more significant, with three studies (27.2%) considered low risk, four (36.4%) assessed as unclear, and four (36.4%) classified as high risk. Regarding the performance bias (blinding of participants and personnel), six studies were assessed as low risk (54.5%), and five (45.5%) were considered high risk. In the detection bias (blinding of outcome assessment), we included five studies (45.5%) under the low-risk tag, two were classified as unclear (18.2%), and four (36.4%) were considered high risk. Most of the studies showed a low risk of bias regarding Attrition bias (incomplete outcome data), with 90.9% of the studies classified as low risk, and 9.1% considered high risk, and Reporting bias (selective reporting), with a 72.7% of low-risk studies, an 18.2% unclear risk, and a 9.1% high-risk studies.

The assessment of every item as well as total score and quality classification are shown in Table 2. The quality of the studies appeared to vary significantly and none of them achieved excellence. Only one study was classified as poor, two as fair, and the rest (8 studies) as good.

**Table 2:** PEDro scores of included studies (reverse chronological order)

Study	Random allocation	Concealed allocation	Groups similar at baseline	Participant blinding	Therapist blinding	Assessor blinding	< 15% dropouts	Intention-to-treat analysis	Between group difference reported	Point estimated and variability reported	Total (0–10)
Turgut et al. [68]	N	N	N	N	N	N	Y	N	Y	Y	3 (Poor)
Bornheim et al. [11]	Y	Y	N	Y	Y	Y	Y	Y	Y	N	8 (Good)
O'Shea et al. [57]	N	Y	N	Y	N	N	Y	N	Y	Y	4 (Fair)
Yi et al. [74]	Y	Y	Y	Y	N	N	Y	N	Y	Y	7 (Good)
Bang and Bong [5]	Y	N	Y	N	N	N	Y	N	Y	Y	5 (Fair)
Smit et al. [64]	N	N	Y	Y	Y	N	Y	N	Y	Y	6 (Good)
Ladavas et al. [47]	N	N	Y	Y	Y	Y	Y	Y	Y	Y	8 (Good)
Brem et al. [12]	N	N	Y	Y	N	Y	Y	Y	Y	Y	7 (Good)
Sunwoo et al. [67]	Y	N	Y	Y	N	Y	Y	N	Y	Y	7 (Good)
Sparing et al. [66]	Y	N	Y	Y	N	N	Y	N	Y	Y	6 (Good)
Ko et al. [46]	Y	N	Y	Y	N	Y	Y	N	Y	N	6 (Good)

### Participants’ characteristics

Main data regarding participants’ characteristics, including sociodemographic variables and clinical characteristics, can be found in Table 3.

**Table 3:** Main data of the included studies

Study	Study design	Experimental conditions	Sample (N)	Stimulation site	Intensity and duration of tDCS	Number of sessions	Assessment measures	Reported results	Improvement in line bisection	Improvement in cancelation task
Turgut et al. [68]	Parallel No randomization No blinding	-Dual tDCS + Optokinetic intervention + Conventional treatment -Conventional treatment alone	32	PPC	1.5–2.0 mA 20 min	8 sessions	- Barthel Index - Body orientation on the wheelchair - Eye, head and trunk orientation (with and without cueing) - Fim/IVAR Early Rehabilitation - Line Bisection Test - The Apples Cancellation Task - The Clock Drawing Test	Combination intervention (dual tDCS + optokinetic + conventional treatment) reduces neglect for ipsilesional spontaneous body orientation and improves performance on the Clock Drawing Test compared to standard treatment	No	No
Bornheim et al. [11]	Crossover within-subjects Case Reports Randomized Double blinding	-a-tDCS + Physical therapy + Occupational therapy + Neuropsychological therapy -Sham tDCS + Physical therapy + Occupational therapy + Neuropsychological therapy	4	M1	2 mA 20 min	20 sessions	- Catherine Bergego Scale - Line Bisection Test - The Star Cancellation Test	Active tDCS improves performance in the four patients in Catherine Bergego Scale, Line Bisection Test, and The Star Cancellation Test, expressed in percentage of change, compared to sham	Yes	Yes
O'Shea et al. [57]	Crossover within-subjects 3 cases study Longitudinal Randomized No blinding	-a-tDCS + Prism adaptation -c-tDCS + Prism adaptation -Sham tDCS + Prism adaptation	3	M1	1 mA 20 min	P1: 3 active and 5 sham sessions P 2: 1 active and 1 sham sessions	-Balloons tests -Coloring, reading and filling in a mock administrative form -Copy Drawing -Letter Cancellation, star cancellation test, object cancellation (dense and sparse search array) -Line Bisection Test -Ota test -The Bells test -Percent correct scores across the tests were combined to compute the overall neglect score for each patient (6 tests for P1; 10 tests for P2 and 3)	Improvement in neglect score after M1 tDCS + prism adaptation compared to sham The improvement in neglect score lasted throughout follow-up (18–46 days)	Yes	Yes
Yi et al. [74]	Parallel Randomized No blinding	-a-tDCS + Conventional physical therapy -c-tDCS + Conventional physical therapy -Sham tDCS + Conventional physical therapy	30	PPC	2 mA 30 min	15 sessions	- Catherine Bergego Scale - Functional Ambulation Classification (basic motor skills) - K-MMSE - Korean Modified Barthel Index - Line Bisection Test - Motor-Free Visual Perception Test - The Star Cancellation Test	Improvement in motor-free visual perception test, line bisection, star cancellation, Catherine Bergego Scale, Barthel Index, and Functional Ambulation Classification in all 3 groups (a-tDCS, c-tDCS, sham) Improvements in the motor-free visual perception test, start cancellation, and line bisection were greater in a-tDCS and c-tDCS compared to sham	Yes	Yes
Bang and Bong [5]	Parallel Randomized No blinding	-tDCS + Feedback training -Feedback training alone	12	PPC	1 mA 20 min	15 sessions	- Barthel index - Line Bisection Test - Motor-Free Visual Perception Test	Improvement in Barthel index, line bisection test and motor-free visual perception test in the two groups Combined tDCS + feedback intervention show greater improvement compared to feedback training alone in all tests	Yes	Not assessed
Smit et al. [64]	Crossover within-subjects No randomized Double blinding	-Dual tDCS -Sham dual tDCS	5	PPC	2 mA 20 min	5 sessions	Behavioral Inattention Test (BIT-C): - Figure and Shape Copying - Letter Cancellation - Line Bisection Test - Line Crossing - Representational Drawing - The Star Cancellation Test	No tDCS-related effects were observed for the BIT subtests	No	No
Làdavas et al. [47]	Parallel Randomized Double blinding	-a-tDCS + Prism adaptation -c-tDCS + Prism adaptation -Sham tDCS + Prism adaptation	30	PPC	2 mA 20 min	10 sessions	Behavioral Inattention Test (BIT-C): - Figure and Shape Copying - Letter Cancellation - Line Bisection Test - Line Crossing - Representational Drawing - The Star Cancellation Test	a-tDCS PPC boosted neglect amelioration after prism adaptation c-tDCS suppresses neglect improvement after prism adaptation	Yes	Yes
Brem et al. [12]	Crossover within-subjects No randomized Double blinding	-Dual tDCS + Conventional cognitive therapy -Sham tDCS + conventional cognitive therapy	1	PPC	1 mA 20 min	6 sessions	Test for Attentional Performance: - Alertness - Covert Attention - Visual field From Neglect-Test German version of BIT: - Cancellation - Copying figures - Line Bisection Test Transfer effects on ADL	Combined intervention (dual tDCS + cognitive therapy) showed greater improvement compared to sham in cover attention, line bisection, and coping ADLs showed improvement at the 3 month follow-up	Yes	No
Sunwoo et al. [67]	Crossover within-subject Randomized Double blinding	-Dual tDCS -a-tDCS -Sham tDCS	10	PPC	1 mA 20 min	3 days Dual tDCS (1) a-tDCS (1)	- Line Bisection Test - The Star Cancellation Test	Significant improvements in line bisection after both dual and single tDCS, but not after sham, being stronger the effect after dual tDCS No significant change in star cancellation	Yes	No
Sparing et al. [66]	Crossover within-subjects No randomized No blinding	-a-tDCS contralesional PPC -c-tDCS contralesional PPC -a-tDCS lesioned PPC -Sham tDCS lesioned PPC	10	PPC	1 mA 10 min	2 days	- Line Bisection Test (computerized version) - Neglect subtest of Test Battery of Attentional Performance’ (TAP)	Both a-tDCS (ipsilesional) and c-tDCS (contralesional) improved line bisection compared to sham No significant changes were detected in neglect subtest of TAP (only a tendency of contralesional c-tDCS)	Yes	Not assessed
Ko et al. [46]	Crossover within-subjects No randomized Double blinding	-a-tDCS -Sham tDCS	15	PPC	2 mA 20 min	2 sessions	- Letter-Structured Cancellation Test - Line Bisection Test - Shape-Unstructured Cancellation Test	a-tDCS showed significant improvement in cancellation tests and line bisection tests compared to sham	Yes	Yes

A total of 152 patients were included in all the studies, with sample sizes from 1 to 32. Three studies included 30–32 patients, four studies included 10–15, and four studies are case reports.

The mean age of the participants was 65.74 years, being 61 women and 91 men. The mean time elapsed after the injury ranged from 48 h to 12.4 years after the stroke. All the studies included post-stroke patients: one study included participants in acute phase (48 h after the event), seven in sub-acute phase (from 20 days to 3.3 months after the stroke), three in chronic phase (between 1 year and 12.4 years), and one study did not specify. In ten studies the participants had right lesions, and one included participants with lesions in both hemispheres.

Regarding the type of stroke, four studies included participants with a diagnosis of ischemic or hemorrhagic stroke, while seven studies did not specify the etiology. In six studies the anatomical location of the lesion was not specified. In the remaining five, different locations are included in the same study (see Table S1 in Supplementary material).

In all studies the diagnosis of neglect was the main criteria for inclusion. However, in six studies it was clearly specified how neglect was assessed and the cutoff point to be included in the sample, while in five studies it was not reported. Regarding the exclusion criteria, in seven studies they were clearly specified, while in four they were not.

### tDCS intervention characteristics

Detailed data of the included studies regarding tDCS intervention protocols can be found in Table S2 (Supplementary material). This table includes whether the stimulation was unilateral or bilateral, the electrodes' position and size, the intensity and density of the applied current, the number and duration of session and complete intervention, tDCS devices, and other treatments applied.

### Unilateral vs. bilateral and location (electrodes position)

In relation to the type of tDCS used, in four studies a dual stimulation (both hemispheres) was performed, while six studies used single stimulation (one hemisphere), and one study combined both types of stimulation (single and dual).

Regarding the location of the electrodes, in most studies (nine) the stimulation was applied over the posterior parietal cortex (PPC), while in two studies it was applied to the primary motor cortex (M1).

In the PPC studies, eight of them applied anodal tDCS in the ipsilesional hemisphere, placing the anode over P4 (six studies), P3 (one study), or P6 (one study). Five studies stimulated the contralesional PPC placing the cathode over P3 (three studies), P4 (one study), or P5 (one study). Four studies included different locations with groups or sub-experiments involving ipsilesional and contralesional tDCS but in single, not dual, stimulation. In single tDCS PPC studies, the reference electrode was positioned in the contralateral supraorbital area in four studies, and on Cz in two studies. One study did not specify the reference electrode position.

In the M1 studies, one of them positioned the anode at C4 and the cathode at FP1 (dual tDCS), while in the other study the anode was placed in the M1 and the reference electrode in the supraorbital area (single tDCS).

A total of five studies carried out dual stimulation, in four of them the electrodes were placed in PPC (anode in P4 and the cathode in P3), while in just one study the target area was M1, with the anode placed at C4 and the cathode at FP1.

### Intensity, electrode size, and density

Regarding the intensity of the applied stimulation, in five studies the stimulation was 2 mA and in another five studies it was 1 mA. In one study, the applied intensity ranged from 1.5 to 2 mA, depending on whether 2.0 mA caused skin irritation.

In relation to the size of the electrode, in four studies the electrode size was 7 × 5 = 35 cm^2^, while in 5 studies the size of the electrode was 5 × 5 = 25 cm^2^. In two studies, no reference was made to the size of the electrode used.

Concerning density, in two studies it was impossible to calculate, since the size of the electrodes was not specified. In the remaining nine, the applied current density was between 0.28 A/m^2^ and 0.8 A/m^2^: 0.8 A/m^2^ (3 studies), 0.4 A/m^2^ (3 studies), 0.28 A/m^2^ (2 studies), and 0.57 A/m^2^ (1 study).

### Number and duration of each session and treatment

Regarding the number of tDCS sessions, in four studies, one or two sessions were carried out; in four studies, between five and 10 sessions were applied; in two studies, 15 sessions were carried out; and in one study the intervention included 20 sessions. In relation to the treatment duration, it ranged from 1 to more than 4 weeks (1 week/5 days, one study; 2 weeks, two studies; 3 weeks two studies; and more than 4 weeks, two studies). There were also four studies that did single sessions.

The majority of the studies (nine studies) applied the stimulation for 20 min, while the duration of the tDCS stimulation was 10 min in one study and 30 min in another one.

### tDCS alone vs. combined

A total of four studies applied tDCS in isolation; in another four, tDCS was applied with another specific intervention aimed at neglect rehabilitation, such as optokinetic task (one study), prism adaptation (two studies), and feedback training (one study). Finally, in three studies, tDCS was applied in combination with a conventional and more general treatment, such as physical therapy, occupational therapy, neuropsychology, or music therapy.

### tDCS device

In four studies, the NeuroConn DC-Stimulator (Ilmenau, Germany) was used, in another four, the device was the Phoresor II Auto Model PM850 (IOMED Inc., Salt Lake City, UT, USA). Only one study used the Standard TCS Starstim (Neuroelectrics, Spain), and another one used the DC-stimulator (Magstim, United Kingdom). In one study, the device employed to apply tDCS was not specified.

### Outcome measures and reported results

The complete list of the tests and tasks used to assess outcome measures can be found in Table S3 (Supplementary material). Detailed data of the included studies regarding outcome measures, times of assessment, and adverse effects assessment, can be found in Table S4. Given the disparity of the study designs (crossover vs. parallel, different kinds of control), the characteristics of the interventions (duration, combination with other techniques), and the limited sample size of most of them, we have not been able to make a quantitative summary of the effects. Nonetheless, we have made a thorough analysis of the results of each assessment in the different studies to make systematic qualitative comparisons between them.

Two studies used the conventional Behavioural Inattention Test (BIT), and the other nine used at least one task from this battery. All the studies used the Line Bisection Test, and a cancellation task (four used the Star Cancellation Test, and seven studies used other cancellation tasks). Most studies included copy or representational drawing tasks. Specifically, two studies used copy drawing, and one applied the Clock Drawing Test. Finally, the Motor-Free Visual Perception Test (MVPT) was used in two studies.

Most studies did not report measures relating to everyday life activities and functional performance. Only three studies reported them using the Barthel Index of Activities of Daily Living, and two applied the Catherine Bergego Scale (CBS). To examine basic motor skills, one study used the Functional Ambulation Classification (FAC), and another study included an assessment of body orientation on the wheelchair, as well as eye, head and trunk orientation with and without cueing.

Moreover, eighteen other tests or tasks (different from the ones mentioned), evaluating one of the domains mentioned above, were applied in the different studies.

Pre- and post-treatment evaluations were carried out in all studies. Nine performed only pre- and post-intervention assessments, and three were follow-up studies. No significant adverse effects were reported in any case, but 4 studies do not report the evaluation of the adverse effects.

Regarding reported results, only one study (with a very limited and chronic sample, *n* = 5; [64] has not found positive results in any neglect assessment test. The rest showed significant greater cognitive and/or functional improvements in tDCS groups compared to control groups, in acute, post-acute, and chronic patients in, at least, one outcome measure (e.g., line bisection task, cancellation task, Barthel Index…). Line bisection and cancellation tests are the most common outcome measures, since all the studies applied a bisection task, and all, except two, applied a cancellation task. Nine studies found improvement in line bisection (two did not, [64, 68], eight and five sessions, respectively), while improvement in cancellation task was found only in five studies out of the nine which assessed it.

Due to the variability in the sample sizes, studies designs, treatment characteristics, and assessment methods it is not possible to establish the best tDCS-based intervention for neglect rehabilitation, being effectives both ipsilesional a-tDCS and contralesional c-tDCS applications. The only thing that seems clear is that tDCS in combination with other interventions focused on neglect rehabilitation (e.g., prism adaptation, optokinetic intervention, feedback training…) is more effective than standard intervention applied alone, as shown in all the combined intervention studies (i.e., [5, 11, 12, 47, 57, 68, 74]).

## Discussion

This systematic review aimed to evaluate current evidence about the efficacy of tDCS as a rehabilitation tool for neglect following a stroke. Eleven studies were included in the review, with a total of 152 patients. Taken together, the results showed that tDCS, used alone or combined with conventional or specific neglect interventions, can improve neglect symptomatology by reducing the visuospatial impairments and ameliorating the deficits related to everyday life activities and functional performance. Nonetheless, the studies reviewed are heterogeneous regarding aspects, such as the number of participants, time since stroke, and study design and methodology. Despite these issues, the therapeutic value of this neuromodulation technique seems very promising, but more research is needed for tDCS to be included in regular neurorehabilitation plans.

### Risk of bias and methodological quality

The risk of bias assessment revealed that most studies had various biases, with selection bias (sequence generation and allocation concealment) being the most common. Many studies failed to design a randomized, blinded study, which could have led to biased results. However, we did not detect attempts to manipulate the impact of the research by reporting incomplete outcome data or selecting specific outcomes.

Concerning the methodological quality, the PEDro scores showed that most of the studies included in the review achieved a good level of quality. However, none reached excellence, and one was classified as poor. The main problems detected were the lack of concealed allocation and the lack of blinding of the therapist and the assessor, even when the studies were not a case report, which may lead to biased treatment outcome estimates. This fact is particularly odd, since the patient blinding is common, but it could be explained by a lack of human resources.

As a result, we can conclude that the risk of bias and methodological quality of the studies analyzed is good enough to take the results into account. Nevertheless, the same biases and missing methodological quality factors repeatedly appear among studies. Hence, some results could unintentionally be slightly misleading.

### Study goals, design, and methodology

Many differences have been detected between studies regarding the control condition and the number of tDCS sessions. While four studies included a control group, the rest used within-subject measures. On the other hand, only four studies conducted 10 or more sessions, with a maximum of 20, and also only 4 studies conducted a follow-up evaluation regarding the long-term maintenance of changes, reporting a maintenance of the improvements obtained after the tDCS intervention. Finally, the sample size is very limited in most of the studies. Therefore, this results in very heterogeneous data that becomes difficult to compare and analyze.

The low number of subjects included in the studies is particularly relevant among all the factors influencing the results. Only three of them had 30 or more participants, with a maximum sample of 32 subjects. The lack of large samples seems to be a recurrent matter in the field of noninvasive brain stimulation, which should raise some concerns not only regarding the use of tDCS in neglect rehabilitation but also in a more general context. Small samples, or insufficient number of participants lead to low statistical power that may imply missing the real effect of the tDCS intervention due to false negative results.

It is also necessary to consider that only 6 studies clearly described the methods used to determine some inclusion criteria objectively; thus, some data cannot be compared and the replicability of some studies becomes harder to achieve.

On the other hand, the results showed that the treatment’s benefits can be sustained for short/medium periods of time [12, 57]. However, only two studies assess the sustained effect (3 month follow-up or more),thus, longer follow-up evaluations are needed to be able to analyze the long-term maintenance of the improvements or determine if additional periodical stimulation sessions are needed to increase the maintenance of the obtained benefits in the short-term.

### Patients’ characteristics

The majority of the patients were in the sub-acute phase, although patients in chronic phase (3 studies) and acute phase (1 study) were also included. Of all the studies, only one with patients in chronic phase did not provide evidence of improvement after the intervention. However, this cannot be explained alone by the chronicity of the pathology, since two other studies also included chronic patients and showed positive outcomes. Therefore, other factors, such as the study design and the intervention characteristics, must have affected the results.

The studies seemed to include both participants who had suffered ischemic and hemorrhagic strokes, although most of the researchers (7 studies) failed to report the etiology of the stroke. In addition, only 3 studies stated the lesion's anatomical location, including the middle cerebral artery (13 patients), the basal ganglia (5 patients), and the posterior cerebral artery (8 patients). Once again, the lack of information becomes a barrier to achieving a comprehensive understanding of the data obtained. Since this information is available in the patients' clinical record, in the majority of cases, researchers must ensure they report it in scientific articles to make comparison and generalization possible.

Concerning the demographic characteristics, 8 studies included more men than women or included only men, resulting in a greater number of men studied. Kleinman et al. already established in 2007 that there were no prevalence or severity differences in hemispatial neglect after stroke between men and women [45]. Nevertheless, both genders should be included equitably in the studies to compensate for such differences and avoid biased results. Hence, we can conclude that there is still a tendency not to enroll enough women in clinical studies. Although we do not believe that researchers do it intentionally, an effort should be made to recruit more women.

On the other hand, the mean age of the participants was 65.74 years, which seems consequent with the fact that more men than women were included in the studies, since men usually have strokes at younger ages than women, and they are more fatal in women. Consequently, the women included in the studies were younger compared to the average age of stroke in women and contributed to lower the average age.

### Intervention characteristics

The studies with positive outcomes used unilateral or bilateral stimulation and were performed either over the PPC or the M1. tDCS on the PPC was the most widely used stimulation protocol and showed improvement of the symptoms when applied as cathodal on the undamaged hemisphere and anodal on the ipsilesional hemisphere. Thus, the results were consistent with the Interhemispheric Rivalry Model previously explained. This data is also consistent with the systematic review conducted by Fisher et al. [26], in which the authors found that proprioceptive alterations were present in various subtypes of neglect, and concluded that neglect resulted from impaired functional connectivity between regions of the brain associated with attention, sensorimotor and visual processes. Proprioceptive functions were related to the premotor areas and prefrontal regions of the cerebral cortex. Then, it seems easy to connect an improvement in the functioning of these regions with an improvement in the symptoms of neglect.

Regarding the combination of different interventions in the neurorehabilitation process, tDCS was mainly applied in combination with either conventional therapy (physical or cognitive) or other specific neglect treatments (i.e., optokinetic intervention, prism adaptation, feedback training), obtaining significantly greater improvement compared to the efficacy of conventional or specific neglect therapy in isolation (i.e., [5, 11, 12, 47, 68, 74]). Thus, it seems that the combination of tDCS with other therapeutic approaches generates a synergistic effect that enhances the benefits of more conventional interventions when applied in isolation.

Regarding the experimental conditions, more than half of the studies (6) only performed single stimulation in the PPC or M1; the rest used dual tDCS (4) or a combination of both (1 study). Dual tDCS was performed by applying anodal stimulation in the lesioned hemisphere and cathodal over the non-lesioned hemisphere. Both types of studies (single and dual) obtained promising data with positive outcomes. Sunwoo et al. [67] was the only study that combined the two conditions and obtained better results with dual than single tDCS. Thus, despite the heterogeneity between studies and the difficulty of comparing them, results suggest that dual tDCS have a stronger effect than single tDCS in neglect rehabilitation.

Differences were found in other aspects related to the intervention characteristics, such as intensity of the stimulation (1 mA, 1.5 mA or 2 mA), size of the electrodes (25 cm^2^ or 35 cm^2^), and current density (between 0.28A/m^2^ and 0.8A/mm^2^). In addition, not all the researchers reported the size of the electrodes, making it impossible to determine the density of the applied current, an essential aspect in the determination of the tDCS effect (e.g., [6]. Therefore, such differences increase the difficulty of comparing results between studies and reaching reliable conclusions.

The duration of the stimulation was one of the more consistent parameters among the studies. The most common duration of the stimulation was 20 min, with only two exceptions, in which it was applied during 10 and 30 min. Conversely, more variability was detected regarding the number of sessions. The studies carried out between 1 and 20 sessions, being more frequent to conduct less than 10 sessions. Although it was impossible to establish a pattern, since the variability was too wide, more prolonged multisession interventions (10 sessions or more) reach more frequently significant results compared to short interventions (8 sessions or less). Furthermore, the complete duration of the treatment was very heterogeneous again, with some studies conducting 1 single session and others lasting weeks, with a maximum time frame of 4 weeks.

Finally, to our knowledge, no research has been undertaken yet using high definition tDCS, and this fact needs to be addressed to increase the knowledge of the real potential of tDCS in neglect rehabilitation.

### Outcome measures

Although all the studies used neuropsychological tests as outcome measures, we identified wide variability in the type of tests used in each research. The most common task used was the line bisection task, which could indicate that this task is perceived as the best measure, with high sensitivity for assessing and detecting changes in neglect symptoms, or that its use can facilitate the comparison between studies. Therefore, any study focused on deepening the knowledge of neglect and its recovery should include this task as an outcome measure. Most of the other tasks used should be analyzed to determine their sensitivity to measure the treatment’s efficacy, since the studies could not always report differences pre-/post-treatment, and it could be due to the insufficient sensitivity of the tests and tasks employed as outcome measures.

Another aspect that needs to be highlighted is the absence in most studies of functional scales to evaluate the disorder’s repercussions on everyday life activities, and the improvement in performance after the intervention. This fact is extremely important, since the final goal of any neurorehabilitation process is to improve the functional independence of the patients and to increase the quality of life of patients, relatives, and caregivers.

Finally, egocentric or allocentric benefits of tDCS should be studied, since their application in different areas could affect the different frames of reference. For example, it is possible that M1 might be better for the egocentric frame and PPC for the allocentric.

### Study limitations

The main limitations in this review are the limited number of studies included, their heterogeneity in terms of methodology and clinically relevant factors, and the possibility that only studies with positive results were published. In addition, some studies do not properly define nor report some essential methodological and clinical aspects, which hinder the generalization of the conclusions.

Across the studies, the most common limitation is the reduced sample size. In addition, the studies differ in the outcome measures employed, and none have sufficiently long follow-up assessments to measure the long-term benefits of tDCS treatment. Moreover, all studies used conventional bipolar electrodes, which implies less focal stimulation. None of the studies used high-definition or multi-site tDCS, which could improve the precision of the stimulation, directing the stimulation to a brain area in a more specific way, which could improve rehabilitation outcomes.

Another constraint for extracting conclusions is the application of tDCS in combination with another type of intervention without an appropriate control group only receiving tDCS. In some studies, it was impossible to attribute the improvement to the application of tDCS, to the other types of treatments also applied, or to the combination of both. It could be advantageous to have more studies, where the application of tDCS could be compared in isolation and together with other types of treatments in homogeneous groups, to be able to analyze the potential for this technique in improving the symptoms of neglect. However, ethical issues come into play, since no patient should be restricted from a treatment whose efficacy has already been proven, such as some specific approaches for neglect rehabilitation, such as prism adaptation. Thus, research must be focused on the efficacy of combined intervention (tDCS + other) in comparison to other proven interventions without tDCS, to find out if there is a synergistic effect that boosts the rehabilitation outcomes.

The heterogeneity of the samples, in terms of clinical characteristics, makes it very difficult to compare the effect of tDCS between patients, due to intragroup variability. For example, we can find cortical and subcortical lesions, hemorrhagic and ischemic lesions, different time of evolution since the injury, different degrees of initial severity of the neglect symptoms, as well as different subtypes of neglect. All of this makes it necessary to select larger and more homogeneous samples to extract powerful and generalizable conclusions.

Finally, a crucial aspect that also needs to be addressed before tDCS can be recommended for neglect is the publication bias. The fact that most studies with negative results are not published makes it very difficult to determine the real potential of the intervention, since we cannot establish how many studies have failed to find significant results. Since the publication bias is deeply rooted in the scientific culture due to many factors from the publication policies to the “publish or perish” imperative in the academic career pursuit, there is not a straightforward solution. Nonetheless, one way to reduce the publication bias is the trial registration [1] and the publication of registered reports [56]. Both methods allow researchers to publish their study protocols before conducting the research and even having them reviewed. Thus, the results of the studies will be published whether they are statistically significant or not. We encourage all researchers to adopt these practices to promote a more transparent and useful scientific knowledge.

## Conclusions

This review found moderate evidence for the efficacy of tDCS in the rehabilitation of hemispatial neglect after a stroke. The results obtained in the studies show that tDCS could be a successful adjuvant therapeutic modality to recover neglect symptomatology, with dual stimulation being more effective. However, the limited number of studies and some studies' design characteristics makes it impossible to draw categorical conclusions at this point.

We are certain that further research is needed to maximize the level of benefit in acute, sub-acute, and chronic stroke patients, including longer follow-ups and neurophysiological measures. In addition, to consolidate non-invasive neuromodulation techniques as therapeutic techniques, it is imperative that a higher level of evidence regarding their efficacy is achieved, by starting to carry out carefully designed studies with larger samples in the immediate future, with the ultimate aim of including tDCS as another useful therapeutical tool, normalizing the use of tDCS in neurorehabilitation settings.

Finally, although this systematic review only examines stroke patients with symptoms of neglect, our recommendations for improving study methodology and reporting of results are relevant to all fields using transcranial electrical stimulation techniques, including tDCS, and should be considered in research with both clinical populations and healthy controls.

## Supplementary Information

Below is the link to the electronic supplementary material.

Supplementary file1 (DOCX 33 KB)
