# Supplementary material for: Transcranial direct current stimulation in neglect rehabilitation after stroke: a systematic review
Source: J Neurol. 2022 Sep 22;269(12):6310–29. doi: 10.1007/s00415-022-11338-x (PMC9618519; doi:10.1007/s00415-022-11338-x)
Supplement: Supplementary file 1 — Supplementary file1 (DOCX 33 KB) [file 415_2022_11338_MOESM1_ESM.docx]

**Transcranial direct current stimulation in neglect rehabilitation after stroke. A systematic review.**

González-Rodríguez, B.* ^a,b^, Serradell-Ribé, N.* ^c^ ^(^[^0000-0002-7572-2476^](https://orcid.org/0000-0002-7572-2476)**^)^**, Viejo-Sobera, R. ^c^ ^(^[^0000-0003-4672-1906^](https://orcid.org/0000-0003-4672-1906)^)^, Romero-Muñoz, JP. ^a,d^ ([0000-0002-3190-1296](https://orcid.org/0000-0002-3190-1296)), Marron, EM. ✉ ^c^ ^(^[^0000-0003-1978-2826^](https://orcid.org/0000-0003-1978-2826)^)^

^a^ Brain Damage Unit, Beata María Ana Hospital. Madrid, Spain.

^b^ Researcher in training. International doctoral school. PhD program in Psychology. Universidad Nacional de Educación a Distancia (UNED). Madrid, Spain.

^c^  Cognitive NeuroLab, Faculty of Health Sciences, Universitat Oberta de Catalunya. Madrid, Spain.

^d^ Faculty of Experimental Sciences. Universidad Francisco de Vitoria. Madrid, Spain.

* Both authors have contributed equally to the paper.

✉ Corresponding author

Elena M. Marron. [emunozmarr@uoc.edu](mailto:emunozmarr@uoc.edu)

Tel. +34 620912976

Plaza de las Cortes, 4. 28014, Madrid

**Journal: Neuropsychology Review**

**Search strategy**

“neglect” OR “hemineglect” OR “hemispatial neglect”

AND

“stroke” OR “cerebrovascular accident” OR “brain ischemia” OR “brain hemorrhage”

AND

“transcranial direct current stimulation” OR “tDCS” OR “non-invasive techniques” OR “noninvasive techniques” OR “non-invasive brain stimulation” OR “noninvasive brain stimulation” OR “NIBS”.

To enhance the sensitivity of our search, we will not include words related to the outcomes of interest.

**Specific main data of the included studies.**

**Table S1.** Main data of the included studies regarding goals, design, and sample characteristics.

| Study | Main study goals | Study design | Experimental conditions | Experimental conditions | Control condition | Sample (N) | Sociodemographic characteristics | Clinical characteristics | Time since stroke |
| --- | --- | --- | --- | --- | --- | --- | --- | --- | --- |
| Turgut et al., 2018 | Efficacy of tDCS in combination with other specific neglect intervention | Parallel  No randomization  No blinding | - Dual tDCS + Optokinetic intervention + Conventional treatment - Conventional treatment alone | Dual tDCS + Optokinetic intervention + Conventional treatment  Conventional treatment alone | Conventional treatment alone | 32 | Women: 11 Men: 21  (16 experimental group, mean age 70,6 (SD. 2.2), 16 control group, mean age 70,6 (SD. 2.2) | Right and left stroke  20 left-sided neglect  12 right-sided neglect  Lesion size 33,9 (SD: 24,9)  25 days since stroke in control, 20 days in experimental (no significant difference between groups) | Sub-acute |
| Bornheim et al., 2018 | Efficacy of tDCS in combination with conventional therapy | Crossover within-subjects  Case Reports Randomized  Double blinding | - a-tDCS + Physical therapy + Occupational therapy + Neuropsychological therapy - Sham tDCS + Physical therapy + Occupational therapy + Neuropsychological therapy | a-tDCS + Physical therapy + Occupational therapy + Neuropsychological therapy  Sham tDCS + Physical therapy + Occupational therapy + Neuropsychological therapy | Sham | 4 | Men: 4  Mean age 68.7 | Right stroke in posterior cerebral artery (n=3), and middle cerebral artery (n=1) Left visuo-spatial neglect  Right-handed patients | Acute |
| O'Shea et al., 2017 | Efficacy of tDCS in combination with other specific neglect intervention | Crossover within-subjects  3 cases study Longitudinal  Randomized  No blinding | - a-tDCS + Prism adaptation - c-tDCS + Prism adaptation Sham tDCS + Prism adaptation | a-tDCS + Prism adaptation  c-tDCS + Prism adaptation Sham tDCS + Prism adaptation | Sham | 3 | Men: 3  Mean age: 58 | Right stroke  Treatment-resistant neglect | Chronic |
| Yi et al., 2016 | Efficacy of tDCS in combination with conventional therapy | Parallel  Randomized  No blinding | - a-tDCS + Conventional physical therapy - c-tDCS + Conventional physical therapy - Sham tDCS + Conventional physical therapy | a-tDCS + Conventional physical therapy  c-tDCS + Conventional physical therapy  Sham tDCS + Conventional physical therapy | Sham | 30 (originally 32, but 2 lost to follow-up because of early discharge) | Women: 9 Men: 21  Mean age: 62.1 | Right stroke  4 hemorrhagic in basal ganglia + 26 ischemic (middle cerebral artery territory alone (n=13), middle cerebral  artery, and anterior cerebral artery territory (n=4), basal  ganglia (n=1), middle cerebral artery and posterior cerebral artery territory (n=4), and middle cerebral artery border-zone (n=4)) | Sub-acute (not clearly specified) |
| Bang et al., 2015 | Efficacy of tDCS in combination with other specific neglect intervention | Parallel  Randomized  No blinding | - tDCS + Feedback training   Feedback training alone | tDCS + Feedback training  Feedback training alone | Feedback training only | 12 | Women: 8 Men: 4  Mean age: 65.8 | Right stroke | Sub-acute |
| Smit et al., 2015 | Efficacy of tDCS in isolation | Crossover within-subjects  No randomized  Double blinding | - Dual tDCS - S - ham dual tDCS | Dual tDCS  Sham dual tDCS | Sham | 5 | Women: 2 Men: 3  Mean age: 64.8 | Left stroke  3 hemorrhagic, 2 ischemic | Chronic |
| Làdavas et al., 2015 | Efficacy of tDCS in combination with other specific neglect intervention | Parallel  Randomized  Double blinding |  | a-tDCS + Prism adaptation  c-tDCS + Prism adaptation  Sham tDCS + Prism adaptation | Sham | 30 | Women: 14 Men: 16  Mean age: 67 | Right stroke  Right-handed patients | Sub-acute |
| Brem et al., 2014 | Efficacy of tDCS in combination with conventional therapy | Crossover within-subjects  No randomized  Double blinding |  | Dual tDCS + Conventional cognitive therapy  Sham tDCS + conventional cognitive therapy | Sham | 1 | Men: 1  Age 72 | Right stroke  Ischemic  Posterior cerebral artery | Sub-acute |
| Sunwo et al., 2013 | Efficacy of tDCS in isolation | Crossover within-subject  Randomized  Double blinding |  | Dual tDCS  a-tDCS  Sham tDCS | Sham | 10 | Women: 6 Men: 4  Mean age: 62.6 | Right stroke  Parietal | Chronic |
| Sparing et al., 2009 | Efficacy of tDCS in isolation | Crossover within-subjects  No randomized  No blinding |  | a-tDCS contralesional PPC  c-tDCS contralesional PPC  a-tDCS lesioned PPC  Sham tDCS lesioned PPC | Sham | 10 | Women: 6 Men: 4  Mean age 57.3 (±16.9 years) | Right stroke  Cortical and/or subcortical vascular lesions  Right-handed patients | Sub-acute |
| Ko et al., 2008 | Efficacy of tDCS in isolation | Crossover within-subjects  No randomized  Double blinding |  | a-tDCS  Sham tDCS | Sham | 15 | Women: 5 Men: 10  Mean age 62.1 (±8.8 years) | Right stroke  Right-handed | Sub-acute |

**Table S2.** Main data of the included studies regarding tDCS intervention characteristics.

| Study | Electrodes position | Electrodes size | Current density (A/m2) | Intensity of tDCS | Duration of tDCS | Number of sessions and tDCS treatment duration | Dual / single tDCS | Experimental conditions (tDCS alone vs. combined) | tDCS device |
| --- | --- | --- | --- | --- | --- | --- | --- | --- | --- |
| Turgut et al., 2018 | Right and left PPC  a-tDCS ipsiles. / c-tDCS contrales.  Right lesioned: a-tDCS P4; c-tDCS P3  Left lesioned: a-tDCS P3; c-tDCS P4 | Not specified | Cannot be calculated due to lack of data | Between 1.5 and 2.0 mA (depending on whether 2.0 mA led to irritation on the skin) | 20 min | 8 sessions  2 weeks | Dual | Combined Dual tDCS + Optokinetic intervention + Conventional treatment  Conventional treatment alone | Not specified |
| Bornheim et al., 2018 | M1 a-tDCS C4; c-tDCS FP1 | 25 cm2 | 0,8 A/m2 | 2 mA  (0,002 A) | 20 min | 20 sessions  4 weeks | Dual | Combined a-tDCS + Physical therapy + Ocupational therapy + Neuropsychological therapy  Sham tDCS + Physical therapy + Ocupational therapy + Neuropsychological therapy | Standard TCS Starstim  (Barcelona, Spain) |
| O'Shea et al., 2017 | M1 a-tDCS over left M1  Reference contralateral supraorbital | 7 x 5 = 35 cm2 | 0,28 A/m2 | 1 mA  (0,001 A) | 20 min | P1 (211 days): Phase A = 4 months (3 sessions sham, 1 active tDCS); Phase 2 = 4 months (2 tDCS, 2 sham) P 2, 3 (77 days): one active tDCS, one sham | Single | Combined a-tDCS + Prism adaptation  c-tDCS + Prism adaptation | DC-stimulator  (Magstim, UK) |
| Yi et al., 2016 | Right and left PPC  a-tDCS P4, reference Cz c-tDCS P3, reference Cz Sham P4 (rPPC), reference Cz | 5 x 5 = 25 cm2 | 0,8 A/m2 | 2 mA  (0,002 A) | 30 min | 15 sessions  3 weeks | Single | Combined a-tDCS + Conventional physical therapy  c-tDCS + Conventional physical therapy  Sham tDCS + Conventional physical therapy | Phoresor II Auto Model PM850  (IOMED Inc., USA) |
| Bang et al., 2015 | PPC a-tDCS over P4 | 5 × 7 = 35 cm2 | 0,28 A/m2 | 1 mA  (0,001 A) | 20 min | 15 sessions  3 weeks | Single | Combined tDCS + Feedback training  Feedback training alone | Phoresor II Auto Model PM850  (IOMED Inc., USA) |
| Smit et al., 2015 | PPC a-tDCS P4; c-tDCS P3 | Not specified | Cannot be calculated due to lack of data | 2 mA  (0,002 A) | 20 min | 5 sessions  5 days | Dual | Alone  Active dual tDCS  Sham dual tDCS | NeuroConn DC-Stimulator  (Ilmenau, Germany) |
| Làdavas et al., 2015 | PPC a-tDCS P6; reference left supraorbital region c-tDCS P5; reference right supraorbital region Sham: 5 patients c-tDCS P5; reference right supraorbital region / 6 patients a-tDCS P6; reference left supraorbital region | 5 × 7 = 35 cm2 | 0,57 A/m2 | 2 mA  (0,002 A) | 20 min | 10 sessions  2 weeks | Single | Combined a-tDCS + Prism adaptation  c-tDCS + Prism adaptation  Sham tDCS + Prism adaptation | Eldith (neuroConn GmbH, Ilmenau, Germany)  Programmable Direct Current Stimulator |
| Brem et al., 2014 | Right and left PPC  a-tDCS P4; c-tDCS P3 | 5 × 7 = 35 cm2 | 0,4 A/m2 | 1 mA  (0,001 A) | 20 min | 6 sessions  4 weeks (only one week of tDCS) | Dual | Combined Dual tDCS + Conventional cognitive therapy  Sham tDCS + Conventional cognitive therapy | NeuroConn DC-stimulator (Eldith, Electro-Diagnostic and Therapeutic Systems GmbH, Ilmenau, Germany) |
| Sunwo et al., 2013 | PPC  Three conditions with 4 electrodes (1) dual-mode: a-tDCS P4, reference left supraorbital area + c-tDCS P3, reference right supraorbital area (2) single-mode: a-tDCS P4, reference the left supraorbital area + sham P3, reference right supraorbital (3) sham: sham both circuits | 5 x 5 = 25 cm2 | 0,4 A/m2 | 1 mA  (0,002 A) | 20 min | 3 days  Dual tDCS (1)  a-tDCS (1) | Single / Dual | Alone  Dual tDCS  a-tDCS  Sham tDCS | Phoresor II Auto ModelPM850 (IOMED, USA) |
| Sparing et al., 2009 | PPC  a-tDCS P3, reference Cz c-tDCS P3, reference Cz a-tDCS P4, reference Cz Sham P4, reference Cz | Active electrode = 25 cm2 | 0,4 A/m2 | 1 mA  (0,002 A) | 10 min | 2 days, with an intersession interval of at least 3h | Single | Alone  a-tDCS contralesional PPC  c-tDCS contralesional PPC  a-tDCS lesioned PPC  Sham tDCS lesioned PPC | NeuroConn GmbH (Ilmenau, Germany) |
| Ko et al., 2008 | PPC  a-tDCS P4, reference left supraorbital area | 5 x 5 = 25 cm2 | 0,8 A/m2 | 2 mA  (0,002 A) | 20 min | 2 sessions 3 days | Single | Alone  a-tDCS  Sham tDCS | Phoresor II Auto ModelPM850 (IOMED, USA) |

**Table S3.** Tests and tasks used in the included studies to assess intervention outcomes.

| *Severity of neglect*  (alphabetic order) | Alertness subtest (intrinsic and phasic) from TAP (Zimmermann & Fimm, 1995) |
| --- | --- |
|  | Balloons Test (serial and popout visual search) (Edgeworth et al., 1988) |
|  | Behavioral Inattention Test, Conventional Subtest (BITC) (Wilson et al., 1987) |
|  | Coloring Task * |
|  | Computerized version of the Line Bisection Test (Fink et al., 2000) |
|  | Copy Drawing Task * |
|  | Copying Figures from BIT (Wilson et al., 1987) ** |
|  | Covert Attention subtest from TAP (Zimmermann & Fimm, 1995) |
|  | Filling in a mock administrative form * |
|  | Letter Cancellation from BIT (Wilson et al., 1987) |
|  | Letter-structured Cancellation Test (Mesulam, 1985) |
|  | Line Bisection Test (LBT) (Schenkenberg et al., 1980; Utz et al., 2011) ** |
|  | Neglect subtest from the Test Battery of Attentional Performance (TAP) (Zimmermann & Fimm, 1995) |
|  | Object Cancellation Task (dense and sparse search array) * |
|  | Ota Task (Ota et al., 2001) |
|  | Reading Task * |
|  | Shape-unstructured Cancellation Test (Mesulam, 1985) |
|  | Star Cancellation Test (SCT) from BIT (Wilson et al., 1987) ** |
|  | The Apples Cancellation Task (Bickerton et al., 2011) |
|  | The Bells Test (Gauthier et al., 1989) |
|  | The Clock Drawing Test from BIT (Wilson et al., 1987) |
|  | The motor-free visual perception test (MVPT) (Colarusso & Hammill, 1996) |
|  | Visual Field subtest from TAP (Zimmermann & Fimm, 1995) |
| *General cognitive impairment* | Mini-mental state examination (K-MMSE) (Folstein et al., 1975) ** |
| *Activities of daily living and functional performance*  (alphabetic order) | Barthel Index (Mahoney & Barthel, 1965) ** |
|  | Beobachtungsleitfaden für räumliche Störungen (BRS) (Neumann et al., 2007) |
|  | Body orientation on the wheelchair (Gossman et al., 2013) |
|  | Eye, head and trunk orientation (with and without cueing) * |
|  | Functional Ambulation Classification (FAC) (Holden, 1984) |
|  | The Catherine Bergego Scale (Azouvi et al., 2003) |
|  | The Functional Independence Measure (FIM) (Keith et al., 1987) ** |

* Ad hoc tasks or adapted from other authors.

** Some authors used language-adapted versions of the test.

**Table S4.** Main data of the included studies regarding outcome measures, and reported results.

| Study | Times of assessment | Assessment measures | Reported results | tDCS adverse effects assessment |
| --- | --- | --- | --- | --- |
| Turgut et al., 2018 | Pre-intervention assessment twice: T1, and T2 four/five days after for baseline stability of neglect symptoms and to control for retest effects, and post-intervention assessment twice: T3, and T4 five to six days after the end of the intervention to assess sustained effects. | - Barthel Index  - Body orientation on the wheelchair  - Eye, head and trunk orientation (with and without cueing)  - Fim/IVAR Early Rehabilitation  - Line Bisection Test  - The Apples Cancellation Task  - The Clock Drawing Test | Combination intervention (dual tDCS + optokinetic + conventional treatment) reduces neglect for ipsilesional spontaneous body orientation and improves performance on the Clock Drawing Test compared to standard treatment. | Not reported |
| Bornheim et al., 2018 | Pre-intervention assessment and every week of the treatment (4 weeks) | - Catherine Bergego Scale  - Line Bisection Test  - The Star Cancellation Test | Active tDCS improves performance in the four patients in Catherine Bergego Scale, Line Bisection Test, and The Star Cancellation Test, expressed in percentage of change, compared to sham. | Not reported |
| O'Shea et al., 2017 | Pre-intervention assessment (multiple times in the 2 weeks before intervention), and post-intervention assessment (multiple times in the 3 weeks after the end of intervention). | - Balloons tests - Coloring, reading and filling in a mock administrative form - Copy Drawing - Letter Cancellation - Line Bisection Test - Object Cancellation (dense and sparse search array) - Ota test - The Bells test - The Star Cancellation Test | Improvement in neglect score after M1 tDCS + prism adaptation compared to sham.  The improvement in neglect score lasted throughout follow-up (18–46 days). | No significant adverse effects. |
| Yi et al., 2016 | Pre-intervention assessment, and post-intervention assessment at the end of the intervention, and 1 week after. | - Catherine Bergego Scale  - Functional Ambulation Classification (basic motor skills)  - K-MMSE  - Korean Modified Barthel Index  - Line Bisection Test  - Motor-Free Visual Perception Test  - The Star Cancellation Test | Improvement in motor-free visual perception test, line bisection, star cancellation, Catherine Bergego Scale, Barthel Index, and Functional Ambulation Classification in all 3 groups (a-tDCS, c-tDCS, sham).  Improvements in the motor-free visual perception test, start cancellation, and line bisection were greater in a-tDCS and c-tDCS compared to sham. | No significant adverse effects. |
| Bang et al., 2015 | Pre-intervention assessment, and post-intervention assessment at the end of the intervention. | - Barthel index  - Line Bisection Test  - Motor-Free Visual Perception Test | Improvement in Barthel index, line bisection test and motor-free visual perception test in the two groups.  Combined tDCS+feedback intervention show greater improvement compared to feedback training alone in all tests. | Not reported |
| Smit et al., 2015 | Pre-intervention assessment, and post-intervention assessment at the end of the intervention, and 3 and 8 weeks after. | BIT-C:  - Figure and Shape Copying  - Letter Cancellation  - Line Bisection Test  - Line Crossing  - Representational Drawing  - The Star Cancellation Test | No tDCS-related effects were observed for the BIT subtests. | No significant adverse effects. |
| Làdavas et al., 2015 | Pre-intervention assessment, and post-intervention assessment at the end of the intervention. | Conventional Behavioral Inattention Test (BIT-C) battery | a-tDCS PPC boosted neglect amelioration after prism adaptation.  c-tDCS suppresses neglect improvement after prism adaptation. | No significant adverse effects. |
| Brem et al., 2014 | Pre-intervention assessment and post-intervention assessment at the end of the intervention, 1 week after, and 3 months after. | Test for Attentional Performance:  - Alertness  - Covert Attention  - Visual field From Neglect-Test  German version of BIT:  - Cancellation  - Copying figures  - Line Bisection Test  Transfer effects on ADL (filled out by the occupational therapist and a family member) | Combined intervention (dual tDCS + cognitive therapy) showed greater improvement compared to sham in cover attention, line bisection, and coping .  ADLs showed improvement at the 3-month follow-up. | No significant adverse effects. |
| Sunwo et al., 2013 | Pre-intervention assessment and post-intervention assessment at the end of the intervention | - Line Bisection Test  - The Star Cancellation Test | Significant improvements in line bisection after both dual and single tDCS, but not after sham, being stronger the effect after dual tDCS.  No significant change in star cancellation. | No significant adverse effects. |
| Sparing et al., 2009 | Pre-intervention assessment and post-intervention assessment at the end of the intervention | - Line Bisection Test ( computerized version)  - Neglect subtest of Test Battery of Attentional Performance’ (TAP) | Both a-tDCS (ipsilesional) and c-tDCS (contralesional) improved line bisection compared to sham.  No significant changes were detected in neglect subtest of TAP (only a tendency of contralesional c-tDCS). | Not reported |
| Ko et al., 2008 | Pre-intervention assessment and post-intervention assessment at the end of the intervention | - Letter-Structured Cancellation Test  - Line Bisection Test  - Shape-Unstructured Cancellation Test | a-tDCS showed significant improvement in cancellation tests and line bisection tests compared to sham | No significant adverse effects. |
